# Population‐Level Assimilated Dietary Responses of Black Bears to Baiting

**DOI:** 10.1002/ece3.74575

**Published:** 2026-10-29

**Authors:** Sarah L. Schooler, Bruce P. Finney, Tyler R. Petroelje, D. Cody Norton, Dean E. Beyer, Jerrold L. Belant

**Affiliations:** ^1^ Department of Fisheries and Wildlife Michigan State University East Lansing Michigan USA; ^2^ Department of Geosciences and Biological Sciences Idaho State University Pocatello Idaho USA; ^3^ Michigan Department of Natural Resources and Environment Marquette Michigan USA

## Abstract

Wildlife feeding through intentional or unintentional practices can result in increased human‐wildlife conflict, disease transmission, population growth, and altered behavior. We quantified how spatial and temporal variation in black bear ( *Ursus americanus* ) bait availability within home ranges influenced black bear diet composition as mediated by sex and age. We tested carbon (δ^13^C) and nitrogen (δ^15^N) isotopes in hair samples from hunter‐harvested bears during pre‐baiting (about 1 May–9 August) and baiting (about 10 August–26 October) periods in Michigan, USA, 2017–2021 and used isotopic mixing models to estimate how diet varied between sexes and with spatially‐varying bait distribution. We used linear models to determine whether intrinsic (i.e., sex, age) and extrinsic (i.e., region, bait quantity and distribution) factors were correlated with δ^13^C and δ^15^N enrichment of black bear hair during pre‐baiting and baiting periods while controlling for corn agriculture, distance from waste accumulation sites, and human population density. Proportion of anthropogenic food in black bear diet was greater during the baiting period, especially for female black bears, but bait density did not influence black bear diet composition. Black bear hair from the Northern Lower Peninsula was more enriched in δ^13^C as compared to the Upper Peninsula and had the greatest δ^13^C and δ^15^N enrichment at about 10 years of age. Bait density and distribution did not influence δ^13^C or δ^15^N enrichment in black bear hair, potentially due to bait availability. Our results demonstrate that anthropogenic food from intentional and non‐intentional sources contributes substantially to black bear diet in Michigan, which may affect body condition, behavior, reproduction, and abundance.

## Graphical abstract

We used individual (e.g., sex, age) and spatial (e.g., bait distribution) predictors to determine how bait impacts black bear diet composition using stable isotope analysis before and during baiting across Michigan, USA. We found that proportion of anthropogenic food in black bear diet was greater during the baiting period, especially for female black bears, and that diet of intermediate‐aged bears was most enriched in δ13C and δ15N. Bait density and distribution did not influence δ13C or δ15N enrichment in black bear hair, potentially due to bait availability.

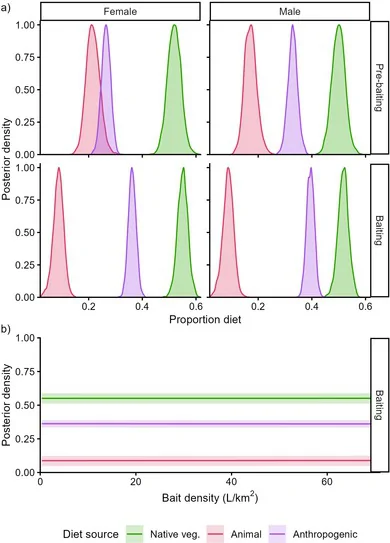

## 1 Introduction

Supplementary feeding of wildlife through unintentional (e.g., agriculture, waste disposal sites) or intentional (e.g., baiting) food subsidies is a global phenomenon (Orams 2002; Newsome, Dellinger, et al. 2015; Murray et al. 2016). Increased food availability from intentional feeding of wildlife may increase survival (Brittingham and Temple 1988) and fecundity (Gould et al. 2021; Field et al. 2026), and therefore population growth rates (Milner et al. 2014) and abundance (Oro et al. 2013) of target species. Supplementary food may also alter species' behavior and habitat use (Steyaert et al. 2014). Therefore, intentional food subsidies may be used by managers to increase species abundance (Orams 2002) or as a diversion to reduce human‐wildlife conflicts, especially in areas or seasons where food availability is limited (Milner et al. 2014). Bait may also be used for scientific research (Stewart et al. 2019) or to increase wildlife viewing or harvest opportunities for target species (Inslerman et al. 2006). However, supplemental feeding of wildlife may have unintended consequences such as increased disease transmission (Milner et al. 2014; Murray et al. 2016), food web alteration (Newsome, Dellinger, et al. 2015), and changes in habitat use (Massé et al. 2014), as well as migratory and hibernation timing (Krofel et al. 2017; Satterfield et al. 2018; Kirby et al. 2019). Supplementary feeding may also have impacts on behavior and abundance of non‐target species (Newsome, Dellinger, et al. 2015; Fležar et al. 2018). Therefore, intentional supplementary feeding of wildlife continues to be controversial (Brown and Cooper 2006; Penteriani and Melletti 2010).

American black bears ( *Ursus americanus* ) forage opportunistically on plants, animals, insects, and anthropogenic foods (Payne et al. 1998; Baldwin and Bender 2009). As black bear diet is primarily driven by food availability (Kirby et al. 2016; Bowersock et al. 2021), black bears in many areas rely on intentional or unintentional food subsidies, especially in years with low natural food availability (Johnson et al. 2015; Pereira et al. 2021). Black bears may have access to unintentional anthropogenic food subsidies through agriculture (Ditmer et al. 2015), waste from landfills or other waste accumulation sites (Rogers et al. 1976), and other human activity (Kirby et al. 2016). Additionally, 12 states in the United States of America allow hunters to use bait (i.e., intentional food subsidies) to attract black bears before and during hunting seasons to increase harvest success (Kirby et al. 2017). Black bear bait may include meat or fish; high‐sugar foods such as cookies, doughnuts, and candy; fruit or vegetables; nuts; and corn, grains, or granola (Kirby et al. 2017; Frawley 2022). Black bears also consume corn bait placed for deer, as deer and black bear baiting and harvest seasons frequently overlap (Wehr et al. 2026). Increased anthropogenic food availability increases amount of anthropogenic food in black bear diets (Kirby et al. 2017), but it is unknown to what extent spatial and temporal variability in distribution of anthropogenic foods, especially bait, influences the amount of anthropogenic food bears consume.

Black bears may perceive anthropogenic food as a high‐quality food source because it is often predictable in space and time, generally requires little energy to acquire, and is more calorie‐dense than other black bear foods (Beckmann and Berger 2003; Baldwin and Bender 2009). Anthropogenic food consumption may vary by sex, reproductive status, age, time of year, and individual (Robbins et al. 2004; Teunissen van Manen et al. 2020). For example, male black bears generally require higher caloric intake than females and may take more risks to acquire high‐quality food (Ditmer et al. 2015). Additionally, as females with cubs may avoid males due to infanticide risk (Ebensperger 1998), male black bears may exclude females with cubs from high‐quality food sources, especially when cubs are young (Ben‐David et al. 2004; Schooler et al. 2025). During hyperphagia, bears have different food quantity and nutritional requirements (Brody and Pelton 1988; Robbins et al. 2007), which may affect seasonal diet composition (Baldwin and Bender 2009). Black bear age and size may also impact diet composition as larger and older bears may be more aggressive, and therefore exhibit increased dominance behaviors, excluding subordinate individuals from high‐quality food sources (Ellis 1995; Zedrosser et al. 2007). However, black bear breeding and reproductive success declines after 8 years of age, which may indicate that intermediate‐aged black bears are more dominant than older black bears (Garshelis and Hellgren 1994; Costello et al. 2009; Johnson et al. 2020).

Stable isotope analysis of black bear hair (δ^13^C and δ^15^N) can be used to estimate plant, animal, and anthropogenic food consumption (Jahren and Kraft 2008; Hopkins and Ferguson 2012). Corn‐based foods are generally higher in δ^13^C, animal‐based foods are generally higher in δ^15^N, and anthropogenic foods have high amounts of both elements. As black bear hair has a known growth rate, seasonal dietary shifts can be determined by sectioning hair based on harvest date to determine diet during different time periods (Jimbo et al. 2020; Finnegan et al. 2023).

We used isotopic analysis of hair samples from black bears legally harvested in Michigan, USA to determine how anthropogenic food availability impacts black bear diet before and during dates when black bear baiting was permitted (“pre‐baiting period” and “baiting period”, respectively). We hypothesized that black bear diet would be affected by bait availability on the landscape as well as sex and age. We predicted that black bears would have higher proportions of anthropogenic food in their diet during the baiting period, as most black bear harvests in Michigan are associated with bait (Frawley 2022). We also predicted that black bear hair would be more enriched in δ^13^C and δ^15^N in areas where hunters reported distributing more bait and, because hunters may be more likely to distribute bait in areas with better road access, hair from bears harvested in areas with higher road density be more enriched in δ^13^C and δ^15^N. As distribution of corn deer bait was banned in the Northern Lower Peninsula (NLP) in 2018, and as deer and black bear baiting periods are similar (Michigan Department of Natural Resources 2026), we predicted that black bear hair during the baiting period would be more enriched in δ^13^C in the Upper Peninsula (UP) as compared to the NLP. We predicted that female black bears would have lower proportions of anthropogenic food in their diet than male black bears due to different energetic requirements and infanticide avoidance. We predicted that hair from intermediate‐age bears would be more enriched in δ^13^C and δ^15^N as compared to hair from younger and older bears, as they may be more socially dominant (Sakiyama et al. 2021).

## 2 Methods

### 2.1 Study Area

We examined black bear diet in the UP and NLP of Michigan, USA using hair samples from harvested black bears during 2017–2021 (Figure 1). Michigan's elevation ranges from 174 m to 890 m above sea level with a mean elevation of 603 m and has a warm‐summer humid continental climate with warm, shorter summers and longer cold winters (Kottek et al. 2006). Temperatures and precipitation vary throughout the state, but 5‐year average (2021–2025) statewide mean, maximum, and minimum temperatures were 8.3°C, 27.5°C, and −10.6°C, respectively (National Oceanic and Atmospheric Administration and Midwestern Regional Climate Center 2026). Five‐year average statewide annual precipitation was 84 cm, and 15‐year average annual statewide snowfall was 202 cm (2006–2020; National Oceanic and Atmospheric Administration 2025; National Oceanic and Atmospheric Administration and Midwestern Regional Climate Center 2026). The NLP (49,133 km^2^) contains approximately 11% of Michigan's population and it is comprised of deciduous forest (28% of NLP area), woody wetlands (20%), and cultivated crops (14%; Kost et al. 2007; United States Census Bureau 2020; Dewitz 2023). The UP of Michigan (43,029 km^2^) contains less than 3% of Michigan's population is heavily forested; landcover is primarily comprised of forests with northern hardwoods (24% of UP area) and conifers (23%); woody wetlands (33%); and bogs (2%) interspersed with agriculture (1%) in the southeastern portion (Kost et al. 2007; United States Census Bureau 2020; Dewitz 2023). Black bears are occasionally observed in the Southern Lower Peninsula but are not common and not permitted to be harvested (Michigan Department of Natural Resources 2008). Bear populations in the NLP and the UP are estimated at 1000–2000 and 7000–11,000 black bears, respectively (D. C. Norton, *unpubl. data*, Mayhew 2019).

**FIGURE 1:**
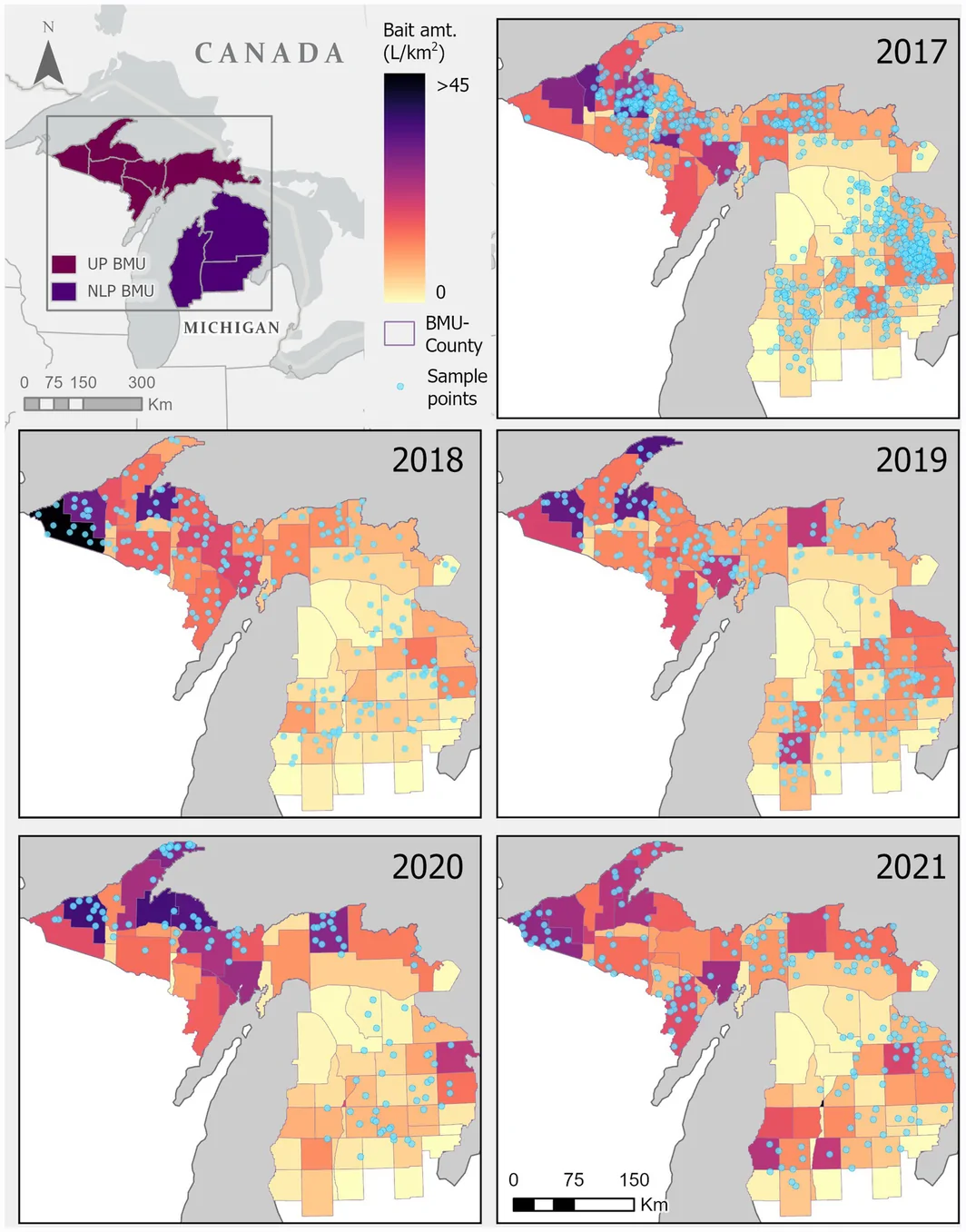
Locations of Upper Peninsula (UP) and Northern Lower Peninsula (NLP) bear management units (BMUs) with amount of bait in liters per square kilometer (L/km^2^) per county‐BMU combination and black bear harvest locations (2.6 km^2^ resolution), Michigan, USA, 2017–2021.

Black bear diet in the Great Lakes region (i.e., Michigan, Minnesota, and Wisconsin) includes vegetation (e.g., grasses [*Gramineae*], sedges [*Carex* spp.], wetland forbs, quaking aspen [ *Populus tremuloides* ]) and ants (*Formicidae*) in spring; ants and fruit (e.g., apples [*Pyrus* spp.], raspberries [*Rubus* spp.], cherries [*Prunus* spp.], blueberries [*Vaccinium* spp.]) in summer; and late‐bearing fruits (e.g., highbush cranberry [ *Viburnum trilobum* ], wild plum [*Prunus* spp.], dogwood [*Cornus* spp.]) and tree mast (e.g., oak [*Quercus* spp.] and American beech [ *Fagus grandifolia* ] in fall; DeBruyn 1992; Payne et al. 1998). Black bears opportunistically feed on animal foods such as snowshoe hares ( *Lepus americanus* ) and white‐tailed deer (especially fawns during spring; *Odocoileus virginianus* ; Payne et al. 1998). Additionally, black bears consume agricultural crops (e.g., corn, fruit [e.g., apples, plums]) and other anthropogenic food from bait piles, waste accumulation sites, and human development (Rogers et al. 1976; Kirby et al. 2017).

Black bears are hunted in the NLP and UP from the second week in September to the third week in October, with exact dates varying by bear management unit (BMU) and year (Michigan Department of Natural Resources 2026). Permits are issued for one of 10 BMUs in Michigan and only male and female black bears without dependent young can be legally harvested (Michigan Department of Natural Resources 2026). On average, 447 and 1304 bears were harvested annually in the NLP and UP respectively during 2017–2021 (Appendix 1; Table A1; Frawley 2022). Successful hunters registered harvested black bears with Michigan DNR where they reported black bear sex, date and location of harvest (2.6 km^2^ resolution [township‐range‐section]; State of Michigan 2022), and Michigan DNR personnel collected guard hair samples collected from the upper middle back of the bear for isotopic analysis and a premolar tooth for age determination (Willey 1974; Frawley 2018; Michigan Department of Natural Resources 2026).

Bear baiting in Michigan is permitted up to 1 month before harvest season through the end of harvest season (Michigan Department of Natural Resources 2026). Permitted black bear bait includes meat, meat products, fish, fish products, fruits, vegetables, bakery, confectionary products, corn, grains, cereal, and candies (excluding chocolate after 2017; Frawley 2022; Michigan Department of Natural Resources 2026). Prior to 2018, all bait types were permitted in unlimited quantities throughout the state, but after 2018, due spread of chronic wasting disease, the use of bear bait that attracts deer or elk (i.e., grains, corn, fruits, vegetables, salt, minerals) was banned entirely in the NLP and was restricted to 2 gal per bait station inaccessible to deer and elk (e.g., in a covered stump, hole, or log) in the UP. Deer baiting was permitted during 15 September–1 January throughout Michigan until 2018, when deer baiting was banned in the NLP. Deer baiting was permitted in the UP during 2017–2021 and could include grains, fruits, vegetables, salts, and minerals, but could not exceed two gallons (7.6 L) with a minimum 3 by 3 m area (Michigan Department of Natural Resources 2018a, 2025).

Michigan DNR provides all bear permit holders the option to report information about bear hunting activity voluntarily via mail and online survey (Frawley 2018, 2022). Questions include hunting location (by county and BMU), hunting success, and hunting methods, including estimated total amount and type of bait distributed (Frawley 2018, 2022). We assumed that hunter survey responses comprised a representative sample of all hunters (Frawley 2022).

### 2.2 Sample Collection and Isotopic Analysis

We used hair samples collected from harvested bears throughout Michigan during 2017–2021 (Figure 1; Appendix 1; Table A1). We assumed that black bear hair grew at a similar rate as brown bear ( *Ursus arctos* ) hair (approximately 0.5 mm per day; Jimbo et al. 2020). Though exact rate of incorporation of diet into hair of black bears is unknown, research on primates (Oelze 2016) and horses (Ayliffe et al. 2004) indicates that dietary isotopes may begin to be detected in mammalian hair as soon as 8 h post‐food consumption. As such, for 2017 samples, we calculated number of days between 30 August and harvest date; for 2018–2021 samples, we calculated number of days between the beginning of baiting period (dependent on year and BMU; between 8 August and 16 August) and harvest date. We then calculated baiting period hair section length by multiplying number of baiting days by 0.5 mm. We selected 2–3 relatively long hairs of similar length per black bear, measured total length, and cut hair into sections, measuring the calculated length from the follicle. Because black bear hair molt occurs in early May, the hair section furthest from the follicle represented spring and summer diet (i.e., the pre‐baiting period), during pre‐baiting and baiting periods while the hair section closest to the follicle represented diet during the baiting period (Jacoby et al. 1999; Mizukami et al. 2005; Teunissen van Manen et al. 2020). We analyzed pre‐baiting and baiting hair samples for each black bear for δ^13^C and δ^15^N using standard isotopic protocols at the University of Wyoming Stable Isotope Facility (2017 samples) and the Interdisciplinary Laboratory for Elemental and Isotopic Analysis located at the Center for Archaeology, Materials, and Applied Spectroscopy on the campus of Idaho State University (2018–2021 samples; Appendix 2; Ben‐David and Flaherty 2012; Finnegan et al. 2023).

### 2.3 Data Extraction

To calculate average black bear home range size, we used black bear Global Positioning Systems (GPS) data during pre‐baiting and baiting periods from the UP of Michigan collected as a part of a long‐term predator–prey study during 2009–2015. We defined the pre‐baiting period as the estimated start of annual hair growth for black bears to the day before the average start date of the baiting period (1 May–9 August; Jacoby et al. 1999; Mizukami et al. 2005; Teunissen van Manen et al. 2020) and the baiting period as the average start and end dates of permitted baiting and harvest across BMUs and years (10 August–26 October). For each period we calculated average 50% home range size for males and females using kernel density estimation (Appendix 1; Table A2; Appendix 2; Gantchoff et al. 2018; Noonan et al. 2019). We opted to use 50% home range area because black bears, especially males and solitary females, can travel long distances for food or mates, and we wanted to determine area of core home range (Hirsch et al. 1999; Gantchoff et al. 2018). For each sample, we used reported harvest location (2.6 km^2^ resolution; Figure 1) as the center point to create a circular buffer of an area equal to sex‐ and period‐specific home range size (i.e., “estimated home range” for each sample), within which we extracted spatial covariate data.

We used hunter survey data on the amount of bait distributed during 2017–2021 (B. Fawley, Michigan DNR, unpublished data) to estimate annual bait density (Appendix 1; Table A1; Appendix 2). Because survey and permit data were collected by county and BMU, which have different boundaries, and we wanted to maximize the resolution of our analysis, we summarized bait data spatially using a combination of county and BMU boundaries (county‐BMU), then adjusted data using reporting rates and number of permits issued to estimate total amount of bait in each county‐BMU combination (Figure 1; Frawley 2022). We then calculated the average bait density within the estimated home range for each sample by weighting the amount of bait in each county‐BMU combination by the proportion of that county‐BMU within sample‐specific home ranges, summing all bait within the home range, then dividing by home range area. To determine how road density impacted black bear diet, we calculated road length divided by home range area for each sample using local and uncoded roads (“tertiary roads”) from the Michigan State linear referencing system (State of Michigan Department of Technology, Management, and Budget and State of Michigan Department of Transportation 2022).

As unintentional food subsidies also impact black bear diet, we controlled for the effects of corn agriculture, human density, and waste accumulation site location on δ^13^C and δ^15^N enrichment in black bear hair. For each sample, we determined the proportion of home ranges covered by corn fields (i.e., corn crop area divided by home range area) using the United States Department of Agriculture (USDA) cropland data layer (USDA National Agricultural Statistics Service (NASS) 2019). We calculated human population density using United States census data shapefiles summarized by blocks (United States Census Bureau 2020), weighting the number of people in each block by the proportion of the block within sample‐specific home ranges, summing blocks within the home range, then dividing this value by home range area. We also calculated average distance within home ranges for each sample to nearest transfer station or compost, solid waste landfill, or processing facility (Michigan Department of Environment, Great Lakes, and Energy 2025a, 2025b, 2025c).

### 2.4 Statistical Analysis

To determine how sex and bait density influenced the proportion of anthropogenic foods in black bear diet, we used isotopic Bayesian mixing models (Moore and Semmens 2008) in R package MixSIAR (Stock et al. 2018; R Core Team 2025) run in the JagsUI package (Kellner 2021) using δ^13^C and δ ^15^N values from black bear hair in the baiting and pre‐baiting periods. As isotopic end results are sensitive to source values, we used previously‐published source values for δ^13^C and δ^15^N of native vegetation (including soft mast and herbaceous plants; Smith et al. 2025) and animal matter (including deer hair and ants; Smith et al. 2025) consumed by black bears from Northern Wisconsin. Given that black bears do not assimilate prey keratin (hair), we added 1.3‰ to δ^13^C of deer hair samples to account for offsets between the isotopic discrimination of hair and muscle tissue (Stephens et al. 2023; Smith et al. 2025). To determine a source value for anthropogenic foods, we calculated the weighted average (based on sample size) of δ^13^C and δ ^15^N source values of corn (Ditmer et al. 2016) and human hair (from Chicago, Illinois; Newsome, Garbe, et al. 2015; Smith et al. 2025; Appendix 1; Table A3). To calculate isotopic values of human food from human hair, we subtracted 2.0‰ and 3.5‰ from mean isotope values for δ^13^C and δ ^15^N, respectively (Newsome, Garbe, et al. 2015) before calculating the weighted average. We corrected each diet group with concentration dependence estimated from mean digestible elemental concentrations for each source value (Hopkins and Ferguson 2012; Kirby et al. 2017). As trophic discrimination factors differ by tissue type, trophic level, and diet composition, and correct usage is critical for accurately determining dietary mixing, we applied source‐specific discrimination factors developed for omnivorous mammals (Kurle et al. 2014). We used fixed effects for sex and estimated density of bear bait (in the baiting period only; Frawley 2022), a random effect for county to control for potential spatial autocorrelation (Semmens et al. 2009) and incorporated process and residual error (Stock and Semmens 2016a). We used three Markov chains with a Monte Carlo run length of 300,000, a burn‐in of 200,000 and a thin rate of 100 (Stock and Semmens 2016b; Stock et al. 2018). We assessed convergence using the Geldman‐Rubin Diagnostic ($\hat{R}$) values and visually checked trace plots and posteriors (Conn et al. 2018), and examined significance using overlap of 95% credible intervals with zero (Gelman et al. 2004).

To assess the effect of period, age, sex, region (UP or NLP), bait density (in the bait period only), and bait distribution (e.g., tertiary road density; Francis et al. 2011) on black bear diet while controlling for corn agriculture, human population density, and distance from waste accumulation sites, we used period‐ and isotope‐specific Gaussian general linear models during pre‐baiting and baiting periods to estimate δ^13^C and δ^15^N in black bear hair. As anthropogenic food is considered high‐quality and may be dominated by bears in better competitive condition, we used a quadratic term for age. As black bear diet composition may vary with natural food availability we controlled for year harvested using a fixed effect (Johnson et al. 2015). We scaled and centered continuous covariates, then calculated pairwise Pearson product–moment correlations, finding no strong correlations among covariates (all |*r*| < 0.7; Fieller et al. 1957). To account for spatial autocorrelation, we included spatial Gaussian Markov random fields for baiting and pre‐baiting linear models (Appendix 2; Anderson et al. 2022; Lindgren and Rue 2015). We fit all four linear models using the R package “sdmTMB” (Anderson et al. 2022). We ensured model convergence and visually checked model residuals for normality, dispersion, and patterns using R package “DHARMa” (Hartig 2022) and examined significance of coefficients by determining if 95% confidence intervals overlapped zero (Gelman and Hill 2007).

## 3 Results

Average black bear home range size was 6.13 km^2^ for females and 125 km^2^ for males during pre‐baiting period, and 44 km^2^ for females and 387 km^2^ for males during the baiting period (Appendix 1; Table A2). On average, 42.1% of hunters issued black bear permits responded to hunter surveys during 2017–2021 (standard deviation [SD] = 2.9%; average *n* responses = 5202 [SD = 123]), and 95.4% (SD = 0.2%) of survey respondents reported using bait to hunt bears (Appendix 1; Table A1). Across the baiting period, average estimated bait density in black bear home ranges was 13.8 and 15.1 L per km^2^ for females and males, respectively (female range = 0.40–73.5 L/km^2^; male range = 0.33–73.5 L/km^2^). Estimated total bait distributed within female and male home ranges was 664 L and 5334 L respectively, or 9.5 L and 76.2 L per day during the 70‐day baiting period.

We analyzed 1449 hair samples (*n* female = 669, *n* male = 728) from the pre‐baiting period and 1432 hair samples (*n* female = 667, *n* male = 719) from the baiting period (Appendix 1; Tables A1 and A4). Samples varied temporally and spatially, though more samples from 2017 were collected and analyzed than in other years (43% of total sample). Potential bear foods (native vegetation, animal, and anthropogenic) were isotopically distinct and δ^13^C and δ^15^N enrichment varied across individuals (δ^13^C range = −27.29‰–−10.64‰; δ^15^N range = 0.15‰–9.11‰; Figure 2; Appendix 1; Table A5).

**FIGURE 2:**
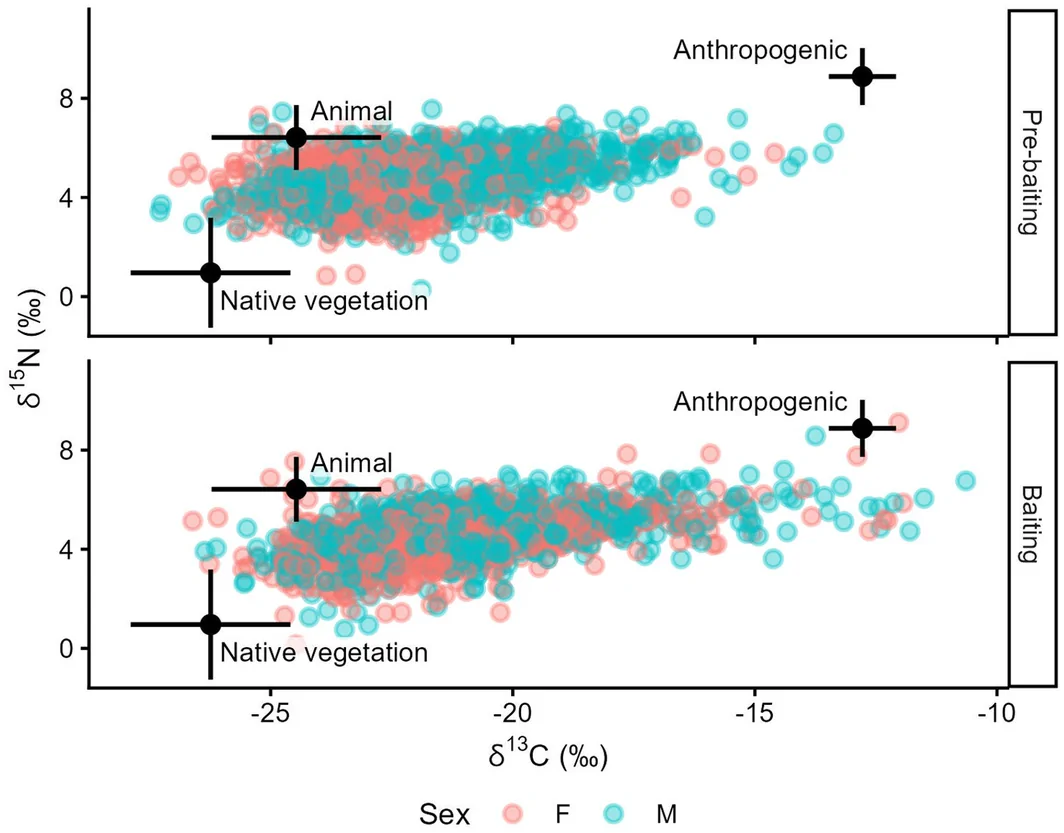
Carbon (δ^13^C, ‰) and nitrogen (δ^15^N, ‰) isotopic signatures of potential forage groups (Kirby et al. 2017) corrected with non‐source‐specific discrimination values (Appendix 2; Table A3) and male and female black bear hair samples in the pre‐baiting (approximately 1 May–9 August) and baiting (approximately 10 August–26 October) periods, Michigan, USA, 2017–2021.

### 3.1 Isotope Models

All Bayesian isotope models converged (all $\hat{R}$ < 1.01) and trace plots demonstrated random scatter around mean values (Gelman et al. 2004; Stock et al. 2018). During the pre‐baiting period, black bear diet included 51.9% native vegetation, 21.6% animal matter, and 26.5% anthropogenic foods, while during the baiting period, diet included 55.0% native vegetation, 8.7% animal matter, and 36.2% anthropogenic foods (Table 1; Figure 3). During the pre‐baiting period, male black bears consumed 2.0% less native vegetation, 4.4% less animal foods and 6.4% more anthropogenic food than female black bears. During the baiting period, bear diet composition did not differ between sexes. Female black bears consumed 9.7% more anthropogenic food during the baiting period than during the pre‐baiting period; male black bears consumed similar amounts of anthropogenic food during both periods. Density of bear bait did not affect black bear diet composition.

**TABLE 1:** Estimated proportional dietary contribution of native vegetation, animal, and anthropogenic foods by period (pre‐baiting [about 1 May–9 August] or baiting [about 10 August–26 October]) and sex (female [F] and male [M]) with continuous slope estimate for the amount of bait (“bait”, included in the baiting model only) from isotopic Bayesian mixing models with means ($\bar{x}$) and 95% credible intervals (2.5%, 97.5%) from Bayesian posteriors, Michigan, USA, 2017–2021.

Period	Predictor		Native vegetation			Animal			Anthropogenic		
			$\bar{x}$	2.5%	97.5%	$\bar{x}$	2.5%	97.5%	$\bar{x}$	2.5%	97.5%
Pre‐bait	Sex	F	0.519	0.47	0.568	0.216	0.171	0.267	0.265	0.232	0.297
		M	0.499	0.451	0.548	0.172	0.13	0.22	0.329	0.294	0.365
Bait	Sex	F	0.550	0.512	0.588	0.087	0.047	0.123	0.362	0.335	0.390
		M	0.517	0.476	0.556	0.088	0.046	0.126	0.395	0.367	0.422
	Bait^a^		0.003	0.001	0.007	0.011	0.013	0.011	−0.017	−0.017	−0.013

^a^ Bait posterior distributions are estimated in isometric log‐ratio space.

**FIGURE 3:**
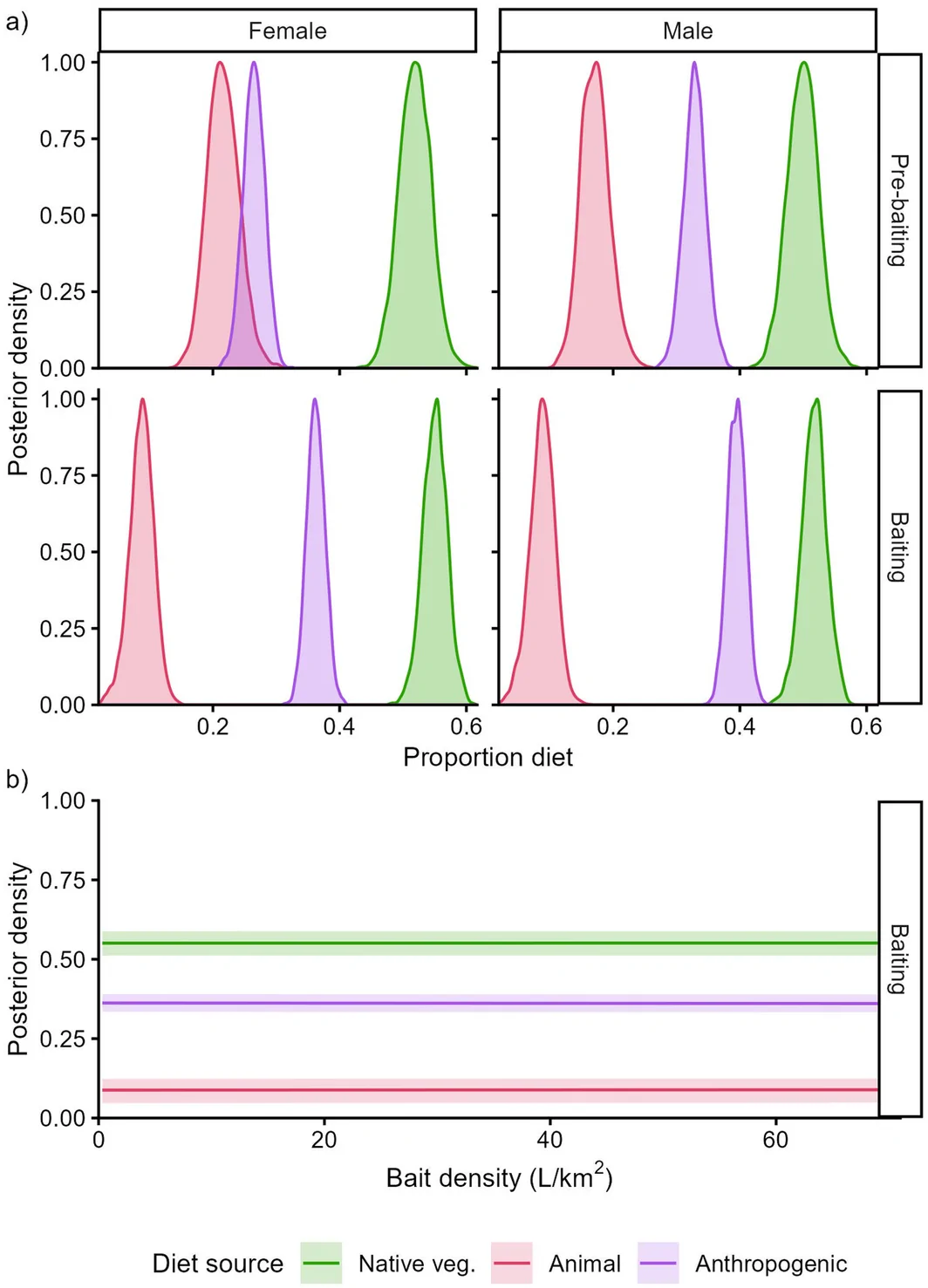
Posterior density estimates from the Bayesian isotope model for black bear diet showing (a) proportion of diet from native vegetation (native veg.), animal food sources, and anthropogenic food sources for female (left) and male (right) black bears during pre‐baiting (approximately 1 May–9 August) and baiting (approximately 10 August–26 October) periods and (b) estimated change in proportion diet with increasing bait density (during baiting period only) with 95% credible intervals, Michigan, USA, 2017–2021.

### 3.2 Gaussian Linear Models

All models converged and residuals showed little evidence of over‐ or under‐dispersion. Male black bear hair was more enriched in δ^13^C and δ^15^N than female black bear hair during both periods (Table 2; Figures 4 and 5). Black bear hair from the UP was less enriched in δ^13^C than from the NLP during both periods, but black bear hair from the UP and NLP was similarly enriched in δ^15^N during both periods. Age had a quadratic relationship with δ^13^C and δ^15^N enrichment in black bear hair; during the pre‐baiting period, δ^13^C enrichment peaked at 10.6 years old (δ^13^C = −20.47‰) and δ^15^N enrichment peaked at 9.7 years old (δ^15^N = 5.37‰). During baiting period, δ^13^C enrichment peaked at 11.2 years old (δ^13^C = −19.33‰) and δ^15^N enrichment peaked at 10.0 years old (δ^15^N = 5.24‰). Density of bear bait within home ranges did not influence δ^13^C or δ^15^N enrichment. Black bear hair was more enriched with δ^13^C with increasing tertiary road density during the baiting period, while tertiary road density had no effect on δ^15^N enrichment in either period.

**TABLE 2:** Table of scaled coefficient estimates (est.) and 95% confidence intervals (CI; 2.5%, 97.5%) from Gaussian general linear models predicting carbon (δ^13^C) and nitrogen (δ^15^N) enrichment in black bear hair during pre‐baiting (about 1 May–9 August) and baiting (about 10 August–26 October) periods with predictors for fixed effects of sex (reference level = female), region (Upper Peninsula [UP]; reference level = Northern Lower Peninsula), age (quadratic effect [age^2^]), proportion of home range covered by corn fields (proportion corn), average distance from waste accumulation sites (waste distance), human population density, total tertiary road length, bait density (for baiting period models only), and year (reference level = 2017); and spatial random effect marginal standard deviation (spatial) and the estimated Matérn range, Michigan, USA, 2017–2021.

	δ^13^C (‰)						δ_15_N (‰)					
	Pre‐baiting			Baiting			Pre‐baiting			Baiting		
Predictor	Est.	2.5%	97.5%	Est.	2.5%	97.5%	Est.	2.5%	97.5%	Est.	2.5%	97.5%
Intercept	−21.376	−21.971	−20.781	−20.081	−20.823	−19.338	5.040	4.674	5.405	4.938	4.668	5.208
Sex												
Male	**0.925**	**0.753**	**1.097**	**0.654**	**0.441**	**0.867**	**0.231**	**0.142**	**0.320**	**0.271**	**0.178**	**0.364**
Region												
UP	**−1.364**	**−2.093**	**−0.636**	**−1.308**	**−2.217**	**−0.398**	−0.394	−0.851	0.064	−0.348	−0.699	0.002
Age	**18.909**	**15.720**	**22.098**	**20.824**	**16.908**	**24.740**	**6.543**	**4.883**	**8.204**	**6.971**	**5.246**	**8.695**
Age^2^	**−14.204**	**−17.378**	**−11.031**	**−14.135**	**−18.025**	**−10.244**	**−6.227**	**−7.890**	**−4.564**	**−6.569**	**−8.306**	**−4.832**
Bait density				−0.001	−0.164	0.163				−0.009	−0.087	0.069
Tertiary road length.	**0.148**	**0.035**	**0.262**	−0.017	−0.202	0.167	0.003	−0.058	0.064	−0.071	−0.159	0.017
Proportion corn	**0.093**	**0.002**	**0.183**	**0.266**	**0.136**	**0.396**	0.008	−0.040	0.056	**0.099**	**0.038**	**0.160**
Waste distance	−0.060	−0.187	0.067	−0.021	−0.189	0.148	**−0.118**	**−0.193**	**−0.043**	**−0.139**	**−0.222**	**−0.056**
Population density	**−0.102**	**−0.204**	**−0.001**	−0.029	−0.176	0.118	0.035	−0.020	0.089	0.053	−0.017	0.122
Year												
2018	−0.543	−0.798	−0.288	−0.932	−1.246	−0.618	0.023	−0.109	0.156	−0.227	−0.364	−0.089
2019	−0.691	−0.925	−0.456	−1.148	−1.438	−0.859	−0.351	−0.473	−0.228	−0.573	−0.701	−0.446
2020	−0.603	−0.946	−0.260	−0.584	−1.008	−0.159	−0.400	−0.578	−0.222	−0.413	−0.600	−0.226
2021	−0.371	−0.662	−0.081	−0.644	−1.002	−0.285	−0.042	−0.194	0.111	−0.215	−0.373	−0.057
Random effects												
Spatial	0.594	0.427	0.827	0.775	0.560	1.073	0.434	0.334	0.563	0.404	0.319	0.513
Matérn	109.449	48.654	246.210	101.490	50.734	203.023	87.578	52.068	147.307	52.493	23.086	119.358

*Note:* Predictors with confidence intervals that did not overlap zero are indicated in bold type.

**FIGURE 4:**
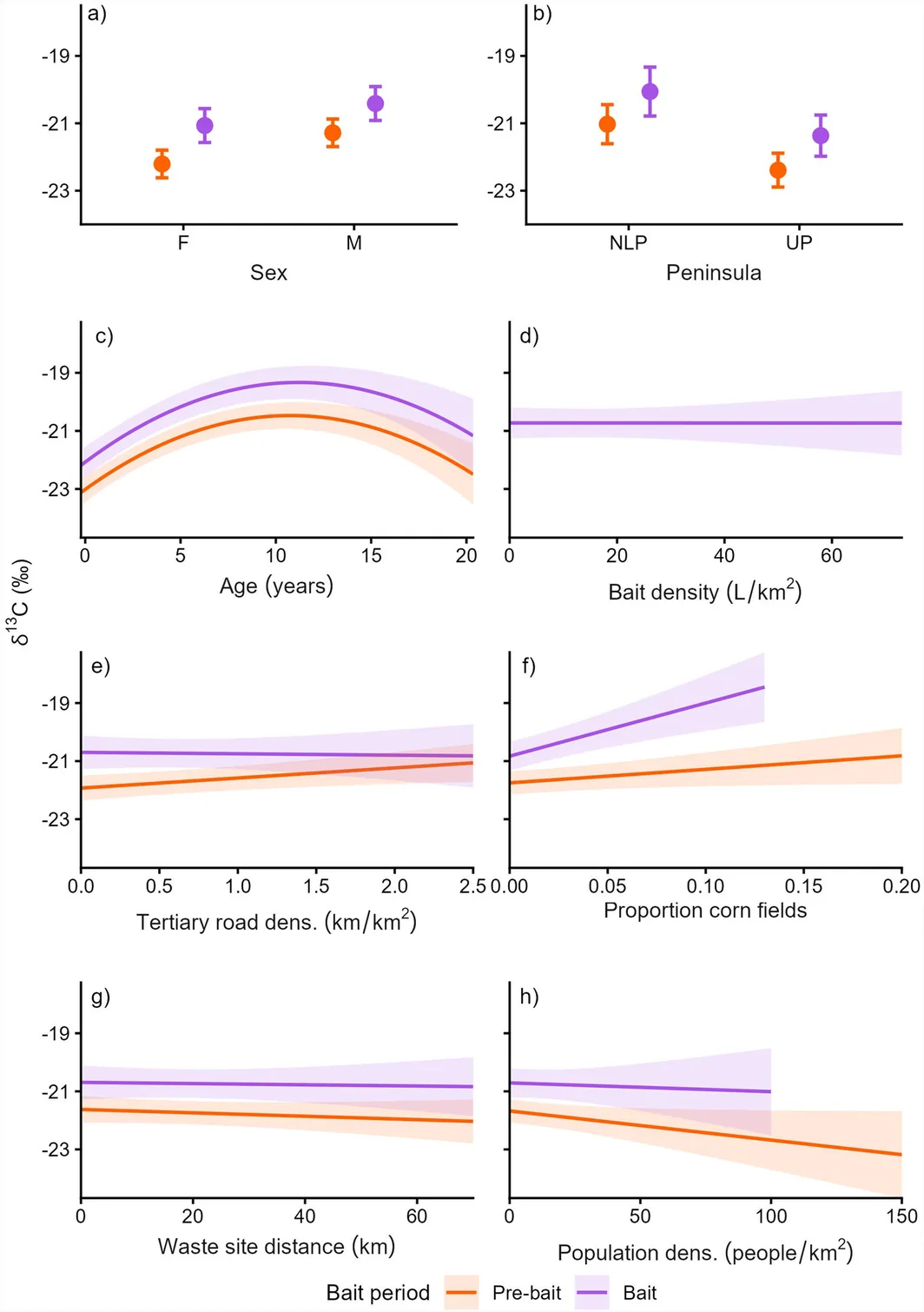
Predicted values from the Gaussian general linear models predicting dietary enrichment of carbon (δ^13^C; ‰) in black bear hair during pre‐baiting (approximately 1 May–9 August) and baiting (approximately 10 August–26 October) periods for predictors of region by sex (a; female [F] and male [M]), peninsula (b; Northern Lower Peninsula [NLP] and Upper Peninsula [UP]), age (c; years), bait density (d; total amount of bait [m^3^]/home range area [km^2^]), tertiary road density (e; total tertiary road length [km]/home range area [km^2^]), proportion of black bear home range covered by corn fields (f; corn; corn field area [km^2^]/home range area [km^2^]), average distance of harvest location from nearest waste accumulation site (g; km), and human population density (h; people/home range area [km^2^]), Michigan, USA, 2017–2021.

**FIGURE 5:**
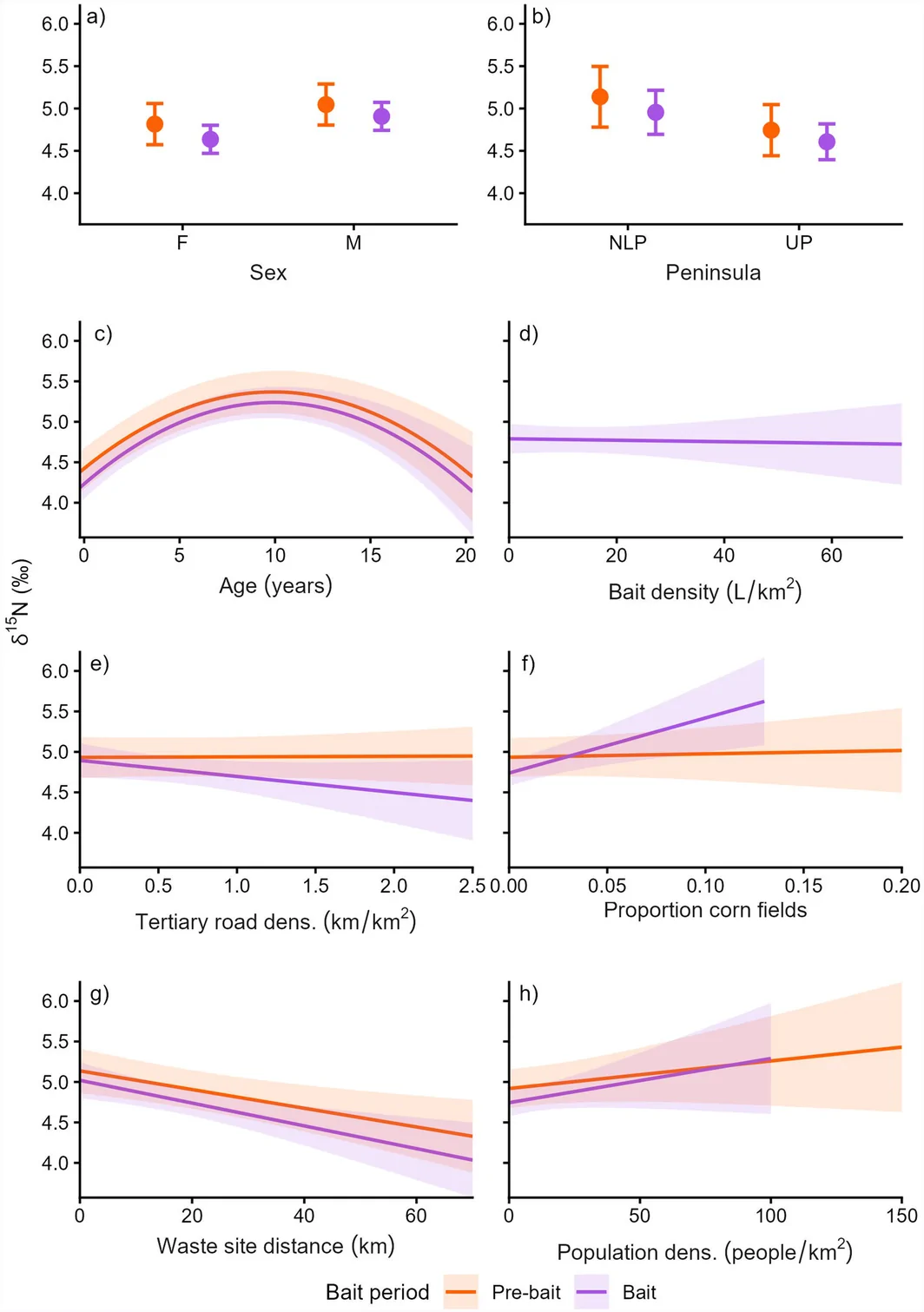
Predicted values from the Gaussian general linear models predicting dietary enrichment of nitrogen (δ^15^N; ‰) in black bear hair during pre‐baiting (approximately 1 May–9 August) and baiting (approximately 10 August–26 October) periods for predictors of region by sex (a; female [F] and male [M]), peninsula (b; Northern Lower Peninsula [NLP] and Upper Peninsula [UP]), age (c; years), bait density (d; total amount of bait [m^3^]/home range area [km^2^]), tertiary road density (e; total tertiary road length [km]/home range area [km^2^]), proportion of black bear home range covered by corn fields (f; corn; corn field area [km^2^]/home range area [km^2^]), average distance of harvest location from nearest waste accumulation site (g; km), and human population density (h; people/home range area [km^2^]), Michigan, USA, 2017–2021.

Enrichment of δ^13^C in black bear hair increased with increasing corn agriculture during both periods, while δ^15^N enrichment increased with increasing corn agriculture during the baiting period (Table 2; Figures 4 and 5). Distance from waste accumulation sites had no effect on δ^13^C enrichment during either period, but δ^15^N enrichment decreased with increasing distance from waste accumulation sites during both periods. Enrichment of δ^13^C in black bear hair decreased with increasing human population density during the pre‐baiting period, but population density had no effect on δ^13^C enrichment during the baiting period or δ^15^N enrichment during either period.

## 4 Discussion

Black bear diet in Michigan consisted of primarily plant foods (45%–60% of diet), followed by anthropogenic (20%–40%), and animal (5%–30%) foods, similar to black bear diet in Wisconsin (Kirby et al. 2017; Smith et al. 2025; Field et al. 2026) and Colorado (Kirby et al. 2016), but different from diets of black bears in Nevada (5%–50% plants; 40%–80% anthropogenic; Teunissen van Manen et al. 2020). Baiting for black bears is permitted in Wisconsin beginning on April 14th through the end of harvest season, but bait must not include animal parts or byproducts, must be completely enclosed (i.e., within a log, hole, or stump), and is limited to 10 gal per bait site (Wisconsin Department of Natural Resources 2021). Black bear baiting is prohibited in Colorado and Nevada (State of Nevada 2011; Colorado Parks and Wildlife 2026). Similar estimated black bear anthropogenic food consumption with varying regulations among states may indicate that black bears in states where baiting is not permitted may consume more unintentional anthropogenic food subsidies such as corn and garbage than in states where baiting is permitted. The similarity of δ^15^N enrichment in black bear hair from bears harvested in Michigan, where bait may include animal parts or products (which are high in δ^15^N), and Wisconsin, where animal parts and products are prohibited in black bear bait is likely because corn and corn‐based foods such as bakery and cereals are also highly enriched in δ^15^N (Hopkins III et al. 2021).

Black bears in Michigan generally consumed more anthropogenic food during the baiting period as compared to pre‐baiting period, but bait density had no effect on δ^13^C or δ^15^N enrichment in black bear hair and did not affect estimated proportions of native vegetation, animal, or anthropogenic foods in assimilated diet. Because bait density was interpolated from survey responses and estimated at the county‐BMU level, the predictor may not accurately reflect the amount of bait available at the finer resolution of estimated black bear home ranges (Frawley 2022). It is possible that the amount of bait available to bears may have exceeded that which they could consume. We estimated 9.5 L and 76.2 L of bear bait per day was distributed within home ranges of male and female black bears, respectively. As captive black bears consume about 7 L of food per day (Rioux et al. 2020), available bait may sufficiently plentiful to sustain multiple black bears with overlapping home ranges, especially in the case of male black bears.

Contrary to our prediction, δ^13^C enrichment in black bear hair increased with tertiary road density during the pre‐baiting, but not the baiting period. Tertiary roads included park roads, two‐track roads, vehicular trails, and neighborhood roads with limited traffic (Federal Highway Administration 2023). Tertiary road density was not correlated with bait density, likely because bait density was calculated at the county‐BMU level and therefore not at a fine enough resolution to represent distribution. Additionally, tertiary road density may not have represented bait distribution well, in part because campgrounds and semi‐rural areas have higher tertiary road density than hunting areas. These areas contain fruit trees, garbage, and bird feeders, with lower risk of human interactions than more highly‐populated areas (Ditchkoff et al. 2006; Merkle et al. 2013; Johnson et al. 2015) and therefore may lead to increased δ^13^C enrichment in black bear diet.

In contrast with our prediction, black bear diet in the NLP was more enriched in δ^13^C than in the UP. Baiting for white‐tailed deer using corn was banned in the NLP in 2018, but the ban was not enforced until 2019 (Michigan Department of Natural Resources 2018b). As most (58%) of our samples were from 2017 to 2018, any potential effect of the deer baiting ban on black bear diet may have been reduced. Additionally, as there is more corn agriculture in the NLP, black bears consumption of corn crops in the NLP may have increased to replace corn from deer bait (Ditmer et al. 2016; United States Department of Agriculture (USDA) National Agricultural Statistics Service (NASS) 2019). Black bear hair from the UP was less enriched in δ^15^N than hair from NLP, which suggests that black bears in the UP consumed more native vegetation, which has less δ^15^N than animal matter, bear bait, or corn (Kirby et al. 2017). Though oak species are more prevalent in the NLP as compared to the UP, the UP has more mixed forests and herbaceous and woody wetlands than the NLP (Dewitz 2023). Therefore certain plant food species (e.g., American beech, cherry, dogwood, wetland forbs, sedges) may be more prevalent in the UP, which could explain the lower δ^13^C and δ^15^N enrichment in black bear hair from the UP (DeBruyn 1992; Kost et al. 2007; Albert et al. 2008).

In contrast with our prediction, male black bears consumed more anthropogenic food than female black bears only during the pre‐baiting period. Males may consume more anthropogenic food than females because body mass may be more important for male black bears, as male reproductive success increases with body size and black bear body size increases with proportion of diet comprised of anthropogenic food (Costello et al. 2009). Due to smaller body size and nutritional costs of pregnancy and lactation, female black bears have different nutritional requirements than males, therefore it may be more optimal for female black bears to consume plant foods over animal or anthropogenic foods (Robbins et al. 2007). Male black bears may also take more risks to obtain perceived higher‐quality food sources (Ditmer et al. 2015). Additionally, larger male black bears may exclude female black bears from prime food sources (e.g., anthropogenic food) due to infanticide risk during spring and summer (Garrison et al. 2007; Reynolds‐Hogland et al. 2024).

During the baiting period, there may be sufficient anthropogenic food available such that female black bears can access preferred food sources without competition (Wehr et al. 2026). Alternatively, during hyperphagia, female black bears may move further and be less risk adverse due to caloric requirements and older‐aged cubs (Landers et al. 1979; Johnson et al. 2015). Intraspecific infanticide, especially during autumn, may not occur as frequently in Michigan as in other populations because black bears are heavily harvested, which may reduce infanticide occurrence (Norton et al. 2018). Also, during the pre‐baiting period (i.e., spring), white‐tailed deer fawns may be an important food source for black bears, but bears may switch to anthropogenic food sources during summer and fall because they are less able to predate deer as fawns increase in size (Zager and Beecham 2006; Kautz et al. 2019).

As we predicted, diet of black bears of intermediate age (about 10 years old) was more enriched in δ^13^C and δ^15^N than bears of other ages during both periods. Black bears attain full body size at 4–6 years old, but attain greatest body mass at 11–12 years and 13–15 years for males and females, respectively (Costello et al. 2009; Nelson et al. 2024). Testosterone levels peak for male black bears at 8 years old and males achieve maximum breeding success at 9–10 years suggesting intermediate‐aged male bears may be more dominant than younger or older bears (Garshelis and Hellgren 1994; Costello et al. 2009). Though female black bears may experience primiparity at as young as 2 years of age, mean age of primiparity likely is 4–8 years of age (Wightman et al. 2022; Reynolds‐Hogland et al. 2022), and intermediate‐aged females are more likely to successfully reproduce (Johnson et al. 2020). Given that intermediate‐aged females are more likely to have cubs, they may be more likely to exhibit dominance behavior of food sources (Herrero 1983; Schooler et al. 2025). Thus, diet of intermediate‐aged black bears may be more enriched in δ^13^C and δ^15^N due to exclusion of other bears from prime feeding locations, such as bait piles or garbage dumps (Rogers et al. 1976).

Enrichment of δ^13^C in black bear hair increased with proportion of home range containing corn crops during the baiting period. Bears typically consume mature corn, which is available in mid‐August in Michigan (e.g., during the baiting period; Lauer 2021). Bears may also consume higher quantities of corn in autumn to help meet caloric requirements of hyperphagia (Brody and Pelton 1988; Baldwin and Bender 2009; Ditmer et al. 2016). Enrichment of δ^15^N in black bear hair was greater near waste accumulation sites during both periods, but distance to waste accumulation sites had no effect on δ^13^C enrichment. Due to the high proportion of meat in the American diet (Newsome, Dellinger, et al. 2015), bears nearer to waste accumulation sites may consume more meat products, which have higher δ^15^N than native vegetation (Schoeller et al. 1986).

As black bear harvest location was reported at 2.6 km^2^ resolution, and because we used a circular buffer of average black bear home range size around harvest location, the landscape variables that we extracted for each bear were coarse estimates of availability. Black bears, especially males and solitary females, can travel long distances for food or mates (Hirsch et al. 1999; Gantchoff et al. 2018). Male black bears in Michigan traveled up to 180 km from their previously established home range in 10 days before returning (Gantchoff et al. 2018). Home range size also may vary with food availability and landscape composition (Mangipane et al. 2018; Penteriani et al. 2021), though generally not with food subsidy quantity (Todorov et al. 2020; Wehr et al. 2026). Also, as all hair samples were from harvested bears which were predominantly harvested using bait (Frawley 2022), anthropogenic food contribution to black bear diet is likely overrepresented compared to the overall black bear population. Similarly, our results on proportion of anthropogenic food in black bear diet during the baiting period may not accurately represent black bear diet through autumn, as our analysis does not reflect dietary changes through and after the hunting season. Too, though we controlled for year as a fixed effect in our linear models, due to sample size limitations, we did not include year as an interaction effect. Because black bears may consume less anthropogenic food in years with increased natural food availability (e.g., acorn mast years; Noyce and Garshelis 1997; Johnson et al. 2015), this may contribute to unexplained variation in our results.

Our assumptions on rates of hair growth and isotopic assimilation into hair may have affected our results. Mammals most likely begin assimilating dietary changes into hair immediately, but change in diet may take up to 40 days to be entirely reflected in hair (Ayliffe et al. 2004; Oelze 2016). The rates of hair growth and isotopic assimilation of food sources into hair may vary by species and season (Brody and Pelton 1988; Ayliffe et al. 2004; Oelze 2016). Because exact assimilation rates are unknown for bears, we assumed rapid dietary assimilation to capture the potential impact of the entire baiting season on black bear diet. Had we assumed a longer assimilation time, we may have found an increased effect of baiting on black bear diet.

Our use of the weighted average of isotopic values of human hair and corn as a source value for the Bayesian isotope model may have biased our estimated proportions of anthropogenic food in black bear diet. Though this method has been used previously to determine unintentional human food subsidies (Smith et al. 2025; Field et al. 2026), actual source values are heavily influenced by proportion of corn and corn syrup in human diets and may not accurately represent anthropogenic foods available to bears. The trophic enrichment factors we chose for our isotopic analysis may have also affected our results on black bear diet composition, as trophic enrichment factors are highly dependent on species, trophic level, and diet source (Stephens et al. 2023). The trophic enrichment factor values we used were from rat hair and varied by diet source (Kurle et al. 2014). We selected trophic enrichment factors from Kurle et al. (2014) because they contributed to successful model convergence and allowed for comparison of diet proportions with Kirby et al. (2017); selection of other trophic enrichment factors may have altered our results (Stephens et al. 2023). However, because we used the same source values and trophic enrichment factors in both pre‐baiting and baiting isotopic models, the inaccuracies in source values and trophic enrichment factors would have a limited effect on our primary hypotheses comparing black bear diet during the pre‐baiting and baiting seasons.

In summary, anthropogenic foods comprised a considerable proportion of black bear diet in Michigan, especially during the baiting period, likely driven by bait and corn availability. Though bait density did not affect proportion of anthropogenic foods in black bear diet, we suggest that this was due to extensive availability of bait, rather than black bears not consuming bait. The resolution and accuracy of the baiting dataset also may have limited our ability to accurately test our hypothesis. Black bear consumption of anthropogenic foods may lead to population increases above carrying capacity, and changes in activity and denning chronology, which may provide increased opportunities for bear hunters due to increased encounter probability and a longer pre‐denning period (Newsome, Dellinger, et al. 2015; Bojarska et al. 2019; Kirby et al. 2019). However, increased populations and shorter denning seasons may also increase likelihood and duration of human‐bear conflicts. Baiting sites may have indirect behavioral and nutritional implications for non‐target species (Newsome, Dellinger, et al. 2015; Candler et al. 2019); wolf‐dog conflicts are more common in Wisconsin as compared Michigan, which may be correlated with an earlier start to baiting season (Bump et al. 2013). Additionally, bait may increase potential pathogen transmission for target and non‐target species (Murray et al. 2016; Fležar et al. 2018); though deer baiting is banned in the NLP to mitigate chronic wasting disease transmission risk, deer also consume black bear bait, which could increase disease transmission risk (Michigan Department of Natural Resources 2018b; Fležar et al. 2018; Candler et al. 2019). Determining the impacts of anthropogenic food availability on black bear diet is warranted when evaluating harvest management decisions and stable isotope analysis of hair is a useful tool to quantify these impacts.

## Author Contributions

**Sarah L. Schooler:** conceptualization (equal), data curation (lead), formal analysis (equal), investigation (lead), methodology (equal), project administration (supporting), writing – original draft (lead), writing – review and editing (equal). **Bruce P. Finney:** data curation (supporting), investigation (equal), methodology (equal), resources (equal), validation (supporting), writing – review and editing (equal). **Tyler R. Petroelje:** funding acquisition (equal), resources (equal), writing – review and editing (equal). **D. Cody Norton:** conceptualization (equal), funding acquisition (equal), supervision (supporting), writing – review and editing (equal). **Dean E. Beyer Jr.:** conceptualization (equal), funding acquisition (equal), methodology (supporting), writing – review and editing (equal). **Jerrold L. Belant:** conceptualization (equal), funding acquisition (lead), methodology (supporting), project administration (lead), supervision (lead), writing – review and editing (equal).

## Funding

This work was supported by the Michigan Department of Natural Resources (WLD2315).

## Conflicts of Interest

The authors declare no conflicts of interest.

## Supporting information


**Appendix 1.** Additional tables.
**Appendix Table A1**. Annual number of permits issued (N permits), responses to harvest surveys (N responses), and male and female hair samples (M samples and F samples respectively) analyzed isotopically by bear management unit (BMU) and region (upper peninsula [UP] and northern lower peninsula [NLP]) from black bear harvest in Michigan, USA, mid‐September–late October, 2017–2021.
**Appendix Table A2**. Number of black bears (*n* bears), mean and standard deviation (std. dev) of number of thinned Global Positioning Systems points (*n* locations) per bear per year and mean and standard deviation of area (km^2^) for 50% home ranges as calculated by kernel density estimation by period (pre‐baiting [1 May–9 August] or baiting [10 August–26 October]) and sex (female [F] and male [M]), Michigan, USA, 2009–2015.
**Appendix Table A3**. Carbon (δ^13^C, ‰) and nitrogen (δ^15^N, ‰) isotopic signatures for source values, digestible concentrations, and discrimination factors (Kurle et al. 2014) for native vegetation (native veg.), animal foods, and anthropogenic foods used in Bayesian isotopic mixing models for diet estimation from hunter‐harvested black bear hair samples with number of samples, means ($\bar{X}$) and standard deviations (SD).
**Appendix Table A4**. Number of black bear hair samples used in analysis for the pre‐baiting (approximately 1 May–9 August) and baiting (approximately 10 August–26 October) periods by sex for each year, Michigan, USA, 2017–2021.
**Appendix Table A5**. Mean ($\bar{X}$) and standard deviation (SD) of carbon (δ^13^C, ‰) and nitrogen (δ^15^N, ‰) isotopic signatures of black bear hair from hunter‐harvested bears by period (pre‐baiting [approximately 1 May–9 August] or baiting [approximately 10 August–26 October]) and sex (female [F] and male [M]) with number of samples analyzed (N), Michigan, USA, 2017–2021.
**Appendix 2**. Additional methods.

## Data Availability

All data and code are available at Dryad/Figshare at https://doi.org/10.5061/dryad.3tx95x6z except for raw hunter survey data and black bear Global Positioning Systems (GPS) points. Hunter survey data are presented as aggregated by year, county, and bear management unit as described in the manuscript to ensure privacy of individuals but anonymized data may be available upon reasonable request from Michigan Department of Natural Resources (dnr-wildlife@michigan.gov). All game animal location data (e.g., black bear GPS data) are legally protected under Sec. 13 (1) (bb) of Michigan Public Act 33 2021; GPS data may be made available upon reasonable request (dnr-wildlife@michigan.gov). Shapefiles of black bear home ranges calculated from GPS points are provided in the uploaded dataset.
