# Supplementary material for: Population‐Level Assimilated Dietary Responses of Black Bears to Baiting
Source: Ecol Evol. 2026 Oct 29;16(11):e74575. doi: 10.1002/ece3.74575 (PMC13719434; doi:10.1002/ece3.74575)
Supplement: Supplementary file 1 [file ECE3-16-e74575-s001.docx]

**Appendix 1: Additional tables**

Appendix Table A1. Annual number of permits issued (N permits), responses to harvest surveys (N responses), and male and female hair samples (M samples and F samples respectively) analyzed isotopically by bear management unit (BMU) and region (upper peninsula [UP] and northern lower peninsula [NLP]) from black bear harvest in Michigan, USA, mid-September–late October, 2017–2021.

| BMU | Region | Year | N permits | N responses | M samples | F samples |
| --- | --- | --- | --- | --- | --- | --- |
| Amasa | UP | 2017 | 469 | 269 | 26 | 25 |
| Baldwin | NLP | 2017 | 149 | 128 | 31 | 32 |
| Baraga | UP | 2017 | 1132 | 400 | 45 | 38 |
| Bergland | UP | 2017 | 951 | 337 | 0 | 2 |
| Carney | UP | 2017 | 482 | 231 | 4 | 3 |
| Drummond Is. | UP | 2017 | 5 | 5 | 2 | 0 |
| Gladwin | NLP | 2017 | 88 | 62 | 9 | 5 |
| Gwinn | UP | 2017 | 868 | 323 | 27 | 22 |
| Newberry | UP | 2017 | 960 | 450 | 51 | 30 |
| Red Oak | NLP | 2017 | 651 | 401 | 165 | 118 |
| Amasa | UP | 2018 | 450 | 217 | 8 | 9 |
| Baldwin | NLP | 2018 | 143 | 118 | 10 | 13 |
| Baraga | UP | 2018 | 1105 | 317 | 15 | 10 |
| Bergland | UP | 2018 | 937 | 303 | 8 | 9 |
| Carney | UP | 2018 | 478 | 235 | 4 | 5 |
| Drummond Is. | UP | 2018 | 5 | 4 | 0 | 2 |
| Gladwin | NLP | 2018 | 83 | 52 | 4 | 2 |
| Gwinn | UP | 2018 | 850 | 291 | 12 | 17 |
| Newberry | UP | 2018 | 905 | 409 | 17 | 20 |
| Red Oak | NLP | 2018 | 632 | 350 | 27 | 24 |
| Amasa | UP | 2019 | 421 | 237 | 8 | 9 |
| Baldwin | NLP | 2019 | 242 | 187 | 17 | 20 |
| Baraga | UP | 2019 | 1154 | 384 | 19 | 11 |
| Bergland | UP | 2019 | 929 | 323 | 3 | 7 |
| Carney | UP | 2019 | 480 | 241 | 6 | 3 |
| Drummond Is. | UP | 2019 | 4 | 4 | 0 | 0 |
| Gladwin | NLP | 2019 | 86 | 69 | 9 | 6 |
| Gwinn | UP | 2019 | 759 | 270 | 22 | 20 |
| Newberry | UP | 2019 | 894 | 424 | 18 | 17 |
| Red Oak | NLP | 2019 | 641 | 393 | 39 | 40 |
| Amasa | UP | 2020 | 440 | 215 | 1 | 4 |
| Baldwin | NLP | 2020 | 243 | 187 | 1 | 2 |
| Baraga | UP | 2020 | 1217 | 299 | 10 | 12 |
| Bergland | UP | 2020 | 963 | 272 | 8 | 3 |
| Carney | UP | 2020 | 482 | 227 | 0 | 0 |
| Drummond Is. | UP | 2020 | 5 | 5 | 0 | 0 |
| Gladwin | NLP | 2020 | 91 | 60 | 1 | 3 |
| Gwinn | UP | 2020 | 789 | 250 | 5 | 7 |
| Newberry | UP | 2020 | 939 | 387 | 6 | 8 |
| Red Oak | NLP | 2020 | 652 | 332 | 15 | 21 |
| Amasa | UP | 2021 | 437 | 224 | 5 | 5 |
| Baldwin | NLP | 2021 | 320 | 240 | 6 | 4 |
| Baraga | UP | 2021 | 1185 | 286 | 3 | 8 |
| Bergland | UP | 2021 | 841 | 294 | 5 | 9 |
| Carney | UP | 2021 | 461 | 200 | 12 | 9 |
| Drummond Is. | UP | 2021 | 6 | 3 | 1 | 1 |
| Gladwin | NLP | 2021 | 107 | 78 | 4 | 3 |
| Gwinn | UP | 2021 | 742 | 253 | 2 | 2 |
| Newberry | UP | 2021 | 999 | 417 | 21 | 21 |
| Red Oak | NLP | 2021 | 713 | 379 | 20 | 29 |

Appendix Table A2. Number of black bears (n bears), mean and standard deviation (std. dev) of number of thinned Global Positioning Systems points (n locations) per bear per year and mean and standard deviation of area (km^2^) for 50% home ranges as calculated by kernel density estimation by period (pre-baiting [1 May–9 August] or baiting [10 August–26 October]) and sex (female [F] and male [M]), Michigan, USA, 2009–2015.

|  |  |  | N locations | |  | Area (km^2^) | |
| --- | --- | --- | --- | --- | --- | --- | --- |
| Period | Sex | N bears | Mean | Std. dev |  | Mean | Std. dev |
| Pre-baiting | F | 19 | 68.39 | 32.95 |  | 6.13 | 3.96 |
| Pre-baiting | M | 25 | 69.06 | 27.61 |  | 125.24 | 225.15 |
| Baiting | F | 17 | 46.96 | 15.67 |  | 43.96 | 85.31 |
| Baiting | M | 20 | 43.61 | 16.66 |  | 387.53 | 958.96 |

Appendix Table A3. Carbon (δ^13^C, ‰) and nitrogen (δ^15^N, ‰) isotopic signatures for source values, digestible concentrations, and discrimination factors (Kurle et al., 2014) for native vegetation (native veg.), animal foods, and anthropogenic foods used in Bayesian isotopic mixing models for diet estimation from hunter-harvested black bear hair samples with number of samples, means (*x̅*) and standard deviations (SD).

|  |  |  |  | δ^13^C (‰) | | | | | |  | | δ^15^N (‰) | | | | | | |
| --- | --- | --- | --- | --- | --- | --- | --- | --- | --- | --- | --- | --- | --- | --- | --- | --- | --- | --- |
| Food | N |  |  | *x̅* | *SD* | *x̅_conc._* | *SD_conc_*_._ | *x̅_disc._* | *SD_disc_*_._ | |  | | *x̅* | *SD* | *x̅_conc._* | *SD_conc_*_._ | *x̅_disc._* | *SD_disc_*_._ |
| Native veg.^1^ | 110 |  |  | -29.64 | 1.61 | 0.47 | 0.03 | 3.4 | 0.2 | |  | | -1.44 | 2.18 | 0.04 | 0.03 | 2.4 | 0.2 |
| Animal^1^ | 52 |  |  | -26.57 | 1.74 | 0.52 | 0.01 | 2.1 | 0.1 | |  | | 2.52 | 1.22 | 0.12 | 0.02 | 3.9 | 0.3 |
| Anthropogenic^2,3^ | 61 |  |  | -16.88 | 0.60 | 0.50 | 0.05 | 4.1 | 0.3 | |  | | 6.08 | 1.11 | 0.05 | 0.01 | 2.8 | 0.2 |

^`^Smith, M. M., D. M. MacFarland, J. L. Price Tack, and J. N. Pauli. 2025. The intentional and unintentional human food subsidy landscape for a large carnivore. Ecology and Evolution 15:e71853.

^2^Ditmer, M. A., D. L. Garshelis, K. V. Noyce, A. W. Haveles, and J. R. Fieberg. 2016. Are American black bears in an agricultural landscape being sustained by crops? Journal of Mammalogy 97:54–67.

^3^Newsome, S. D., H. M. Garbe, E. C. Wilson, and S. D. Gehrt. 2015. Individual variation in anthropogenic resource use in an urban carnivore. Oecologia 178:115–128.

Appendix Table A4. Number of black bear hair samples used in analysis for the pre-baiting (approximately 1 May–9 August) and baiting (approximately 10 August–26 October) periods by sex for each year, Michigan, USA, 2017–2021.

| Year | Pre-baiting | | Baiting | |
| --- | --- | --- | --- | --- |
|  | Female | Male | Female | Male |
| 2017 | 275 | 357 | 272 | 347 |
| 2018 | 111 | 105 | 111 | 105 |
| 2019 | 133 | 140 | 133 | 141 |
| 2020 | 59 | 47 | 60 | 47 |
| 2021 | 91 | 79 | 91 | 79 |

Appendix Table A5. Mean (*x̅*) and standard deviation (SD) of carbon (δ^13^C, ‰) and nitrogen (δ^15^N, ‰) isotopic signatures of black bear hair from hunter-harvested bears by period (pre-baiting [approximately 1 May–9 August] or baiting [approximately 10 August–26 October]) and sex (female [F] and male [M]) with number of samples analyzed (N), Michigan, USA, 2017–2021.

|  |  |  | δ^13^C (‰) | |  | δ^15^N (‰) | |
| --- | --- | --- | --- | --- | --- | --- | --- |
| Period | Sex | N | *x̅* | SD |  | *x̅* | SD |
| Pre-baiting | F | 669 | -22.31 | 1.79 |  | 4.66 | 0.96 |
| Pre-baiting | M | 728 | -21.54 | 2.22 |  | 4.87 | 0.96 |
| Baiting | F | 667 | -21.20 | 2.29 |  | 4.50 | 1.04 |
| Baiting | M | 719 | -20.67 | 2.39 |  | 4.76 | 0.99 |

**Appendix 2: Additional methods**

*Isotopic Analysis*

We cleaned each sectioned hair sample of debris and washed with a 2:1 chloroform-ethanol solution, rinsed with distilled water, then dried at 60°C for at least 48 hours (Hobson et al., 2000). We cut hair samples in small pieces (about 1 mm) and weighed each for mass spectrometry using standard target weights for biological samples (0.5 mg; Thomas et al., 2022). We determined δ^13^C and δ^15^N compositions using an isotope ratio mass spectrometer (Thermo Delta V Advantage with ConFlo IV interface, Thermo Fisher Scientific, Waltham, Massachusetts, USA) and an elemental analyzer (Costech, Valencia, California, USA) at the Interdisciplinary Laboratory for Elemental and Isotopic Analysis, Center for Archaeology, Materials, and Applied Spectroscopy at Idaho State University. Precision for both measurements was ±0.2‰. All δ^13^C and δ^15^N are reported conventionally as ratios relative to the international isotope standards of Vienna Pee Dee limestone (carbon) and atmospheric N_2_ (nitrogen) as follows (Ben-David & Flaherty, 2012):

$$\delta X={10}^{3}*\left[ \frac{R_{sample}}{R_{standard}}-1 \right]$$

where X is the element in its heavy form (^13^C or ^15^N) and R are the ratios of heavy to light isotopes in the sample and standard.

*Home range determination*

Black bears were captured using modified Aldrich foot snares and culvert traps, immobilized, and fit with Global Positioning Systems (GPS) radiocollars (Lotek Wireless, Newmarket, Ontario, Canada) with leather breakaway links (Garshelis & McLaughlin, 1998) and a sampling interval of 15 minutes (Gantchoff et al., 2018; Johnson & Pelton, 1980).

We monitored 45 individual black bears (19 female, 26 male) during 2009–2015 in the Upper Peninsula of Michigan, and as some were monitored for multiple years, we treated each year of data as independent, totaling 92 bear-years of data (Appendix 2 Table A1). We removed data after black bears entered dens in the fall, based on visual identification of clustering in GPS points (Waller et al., 2012). We thinned bear data to one observation per day to minimize spatiotemporal autocorrelation then calculated 95% and 50% home ranges for each animal, year, and season (pre-baiting [1 May–9 August] or baiting [10 August–26 October]) using kernel density estimation in program R using package “ctmm” (Fleming & Calabrese, 2021; Noonan et al., 2019; R Core Team, 2025). We did not use bear-year-season combinations with less than 15 days of relocation data to ensure accurate home range estimation (Noonan et al., 2019). We calculated the area for each home range, then determined the average home range size across bears for each sex and season. We opted to use 50% home range area for modeling because black bears, especially males and solitary females, can travel long distances for food or mates (Gantchoff et al., 2018; Hirsch et al., 1999). Home range area estimates were then used as area for a circular buffer around the center of the harvest location (resolution = 2.6 km^2^) to extract spatial covariates.

*Bait calculations*

If hunters recorded that they used bait and which type but did not provide an estimated amount, we used the average amount of that bait type used within that county-BMU. If hunters hunted in multiple counties, we split the bait used evenly among counties. We then adjusted for varying survey reporting rates among years and BMUs by multiplying the hunter and bait values by the inverse of the proportion of surveys returned to total permits issued for each BMU and year (assuming that every person issued a permit hunted; Frawley, 2022). We calculated total density of hunters and bait within a black bear’s home range by multiplying the bait amount and number of hunters using bait by the proportion of the BMU-county polygon covered by the home range, summing all values within a home range, then dividing by the home range area.

*Spatial Autocorrelation Model Fitting*

To optimize model fitting, we first created an interior border by building a nonconvex hull using our data points with an extended curvature (i.e., convex parameter) of -0.06, a minimum reentrant coverture (i.e., concave parameter) of -0.05 with a gridded resolution of 2,600 longitudinally and 2,200 latitudinally using the R package “fmesher”(Lindgren, 2024). We then created a 2-D mesh with an inner border of the convex polygon, an offset of 5, a maximum edge of 150, a minimum angle of 22, and a minimum allowed distance between points (i.e., cutoff) of 22 (Anderson et al., 2022; Lindgren, 2024; Lindgren & Rue, 2015).

**Appendix 2 Works Cited**

Anderson, S. C., Ward, E. J., English, P. A., & Barnett, L. A. K. (2022). sdmTMB: An R package for fast, flexible, and user-friendly generalized linear mixed effects models with spatial and spatiotemporal random fields. *BioRxiv*, *2022.03.24.485545*. https://doi.org/10.1101/2022.03.24.485545

Ben-David, M., & Flaherty, E. A. (2012). Stable isotopes in mammalian research: A beginner’s guide. *Journal of Mammalogy*, *93*(2), 312–328. https://doi.org/10.1644/11-MAMM-S-166.1

Fleming, C. H., & Calabrese, J. M. (2021). *ctmm: Continuous-time movement modeling* (Version 1.2.0) [R package].

Frawley, B. J. (2022). *2021 Michigan black bear hunter survey* (Wildlife Division Report No. 3705; p. 52). Michigan Department of Natural Resources.

Gantchoff, M., Wang, G., Beyer, D., & Belant, J. (2018). Scale-dependent home range optimality for a solitary omnivore. *Ecology and Evolution*, *8*(23), 12271–12282. https://doi.org/10.1002/ece3.4690

Garshelis, D. L., & McLaughlin, C. R. (1998). Review and evaluation of breakaway devices for bear radiocollars. *Ursus*, *10*, 459–465.

Hirsch, J. G., Bender, L. C., & Haufler, J. B. (1999). Black Bear, *Ursus americanus*, movements and home ranges on Drummond Island, Michigan. *The Canadian Field-Naturalist*, *113*(2), 221–225. https://doi.org/10.5962/p.358559

Hobson, K. A., McLellan, B. N., & Woods, J. G. (2000). Using stable carbon (δ13C) and nitrogen (δ15N) isotopes to infer trophic relationships among black and grizzly bears in the upper Columbia River basin, British Columbia. *Canadian Journal of Zoology*, *78*(8), 1332–1339. https://doi.org/10.1139/z00-069

Johnson, K. G., & Pelton, M. R. (1980). Prebaiting and snaring techniques for black bears. *Wildlife Society Bulletin (1973-2006)*, *8*(1), 46–54.

Lindgren, F. (2024). *fmesher: Triangle meshes and related geometry tools* (Version 0.2.0) [R package].

Lindgren, F., & Rue, H. (2015). Bayesian Spatial Modelling with R - INLA. *Journal of Statistical Software*, *63*(19). https://doi.org/10.18637/jss.v063.i19

Noonan, M. J., Tucker, M. A., Fleming, C. H., Akre, T. S., Alberts, S. C., Ali, A. H., Altmann, J., Antunes, P. C., Belant, J. L., Beyer, D., Blaum, N., Böhning-Gaese, K., Cullen, L., de Paula, R. C., Dekker, J., Drescher-Lehman, J., Farwig, N., Fichtel, C., Fischer, C., … Calabrese, J. M. (2019). A comprehensive analysis of autocorrelation and bias in home range estimation. *Ecological Monographs*, *89*(2), 1–21. https://doi.org/10.1002/ecm.1344

R Core Team. (2025). *R: a language and environment for statistical computing* (Version 4.5.2) [Computer software]. R Foundation for Statistical Computing. https://www.r-project.org/

Thomas, S. N., French, D., Jannetto, P. J., Rappold, B. A., & Clarke, W. A. (2022). Liquid chromatography–tandem mass spectrometry for clinical diagnostics. *Nature Reviews. Methods Primers*, *2*(1), 96. https://doi.org/10.1038/s43586-022-00175-x

Waller, B. W., Belant, J. L., Young, B. W., Leopold, B. D., & Simek, S. L. (2012). Denning chronology and den characteristics of American black bears in Mississippi. *Ursus*, *23*(1), 6–11.
